# Screening and Evaluation of Novel Compounds against Hepatitis B Virus Polymerase Using Highly Purified Reverse Transcriptase Domain

**DOI:** 10.3390/v12080840

**Published:** 2020-07-31

**Authors:** Eriko Ohsaki, Keiji Ueda

**Affiliations:** Division of Virology, Department of Microbiology and Immunology, Osaka University Graduate School of Medicine, 2-2 Yamada-oka, Suita, Osaka 565-0871, Japan; eohsaki@virus.med.osaka-u.ac.jp

**Keywords:** hepatitis B virus, polymerase, reverse transcriptase, high-throughput screening

## Abstract

Hepatitis B virus (HBV) polymerase seems to be very hard to express and purify sufficiently, which has long hampered the generation of anti-HBV drugs based on the nature of the polymerase. To date, there has been no useful system developed for drug screening against HBV polymerase. In this study, we successfully obtained a highly purified reverse transcriptase (RT) domain of the polymerase, which has a template/primer and substrate binding activity, and established a novel high-throughput screening (HTS) system using purified RT protein for finding novel polymerase inhibitors. To examine whether the assay system provides reliable results, we tested the small scale screening using pharmacologically active compounds. As a result, the pilot screening identified already-known anti-viral polymerase agents. Then, we screened 20,000 chemical compounds and newly identified four hits. Several of these compounds inhibited not only the HBV RT substrate and/ template/primer binding activity, but also Moloney murine leukemia virus RT activity, which has an elongation activity. Finally, these candidates did show to be effective even in the cell-based assay. Our screening system provides a useful tool for searching candidate inhibitors against HBV.

## 1. Introduction

Hepatitis B virus (HBV) is a major risk factor of liver cirrhosis and hepatocellular carcinoma in humans. Approximately two billion people are infected with HBV and 350 million individuals worldwide are chronically infected with HBV [1]. Although vaccination and anti-HBV drugs such as interferons and nucleos(t)ide analogs are available for prevention and treatment, they possess limited efficacy and frequently cause severe side effects [2,3,4]. Thus, virus elimination from chronically infected patients is quite a serious and difficult issue. Furthermore, it has seemed practically impossible to eliminate HBV even from acutely infected patients due to features unique to HBV [5,6].

Needless to say, viral polymerase is an ideal target for repressing viral genome amplification. For drug development targeting HBV polymerase (HBV Pol), it is important to aim for control of the viral dose in infected patients to prevent disease progression. A recent study has demonstrated that the drug combination of non-nucleos(t)ide reverse transcriptase inhibitor (NNRTI) and nucleos(t)ide reverse transcriptase inhibitor (NRTI) showed the stronger synergistic inhibitory effect against even multi-NRTI-resistant HIV-1 strains [7]. However, to date, there has been no NNRTI against HBV, and thus the development of novel NNRTI must be an important issue. Recently, Voros and co-workers presented an efficient strategy for purifying the recombinant HBV Pol domain and performing structural and biophysical characterizations [8]. However, it has remained extremely difficult to obtain a sufficient amount of highly pure, full-length HBV Pol, which accounts for the relative paucity of systems for detecting HBV Pol activity.

In this study, we aimed to establish an HTS system to screen novel polymerase inhibitors. For this purpose, we attempted the expression and purification of the reverse transcriptase (RT) domain of HBV Pol (HBV RT) using an *E. coli* expression system and we successfully obtained highly pure RT protein in excess of that previously reported. The purified RT protein did retain specific binding activity with a template/primer (T/P) and a substrate, although it did not show any polymerizing activity. The purified protein was then utilized to establish an assay system for detecting the T/P and substrate binding activity of HBV RT. The function and structure of the T/P and substrate binding domain of various polymerases are highly conserved across species [9,10,11,12,13], and T/P and substrate binding are the first essential step of the polymerase reaction [14,15]. Using the present system, we screened a chemical library from a manufacturer (LOPAC^®^; Sigma, St. Louis, MO, USA) and one from the Center for Drug Discovery, Design, and Development at Osaka University. We identified gossypol, suramin, and NF023, among other compounds, from the manufacturer’s library. From the Osaka University chemical library, we obtained four hits on compounds capable of inhibiting RT-specific T/P and substrate binding activity. To evaluate the inhibitory effects of hit compounds on HBV DNA replication, cell-based assays were performed by using an HBV-producing cell line, HB611 and NTCP-expressing HepG2 cell line.

## 2. Materials and Methods

### 2.1. Plasmids and Antibodies

For construction of a tagged HBV RT, we used the pQE-TriSystem His-Strep 2 vector (QIAGEN, Germantown, MD, USA). To insert an HBV RT fragment spanning from 1039 to 2070 nt, 347 to 690 amino acids, strain adr4 (genotype C) (GenBank accession: X01587) into the vector, the RT fragment was amplified by PCR with primers designed with a Pvu II site at both ends (Forward: 5′-ttcagctggaggactggggaccctgcac-3′, Reverse: 5′-ttcagctgttgccgggcaacggggtaaa-3′ (Pvu II sites are underlined)) and an HBV RT expression vector, pQE-His-Strep2-RT, was constructed by the insertion of the RT fragment into the Pvu II site of the pQE-TriSystem His-Strep 2 vector (QIAGEN), which had a Strep-tag- and an 8× His-tag-coding sequence at the 5′- and at the 3′-ends of the multi cloning site, respectively. A mutant HBV RT gene in which a highly conserved YMDD motif had been changed to AAAA was synthesized (Gene Art^®^, Thermo Fisher Scientific, Rochester, NY, USA), and this mutant was then cloned into the same vector as the wild type. For the expression of a control protein, His-SUMO (Small Ubiquitin-like Modifier), pE-SUMOpro Amp (LifeSensors, Malvern, PA, USA) was used.

### 2.2. Protein Expression and Purification

For the expression of strep-RT-his8 (HBV RT) or his-SUMO protein in *E. coli*, pQE-His-Strep2-RT or pE-SUMOpro was introduced into an *E. coli* strain, Rosetta-gami™ B (DE3) pLysS (Novagen, Wisconsin, USA). The transformed *E. coli* was pre-cultured to a density of 0.6 to 0.8 at 600 nm, and then the transformants were induced for protein expression with 1 mM isopropyl-β-d-thiogalactopyranoside (IPTG) at 37 °C and harvested after 6 h. Protein purification was performed according to methods used in a previous study [8]. In brief, the *E. coli* pellets were lysed in lysis buffer (20 mM Tris-HCl [pH 8.0], 500 mM NaCl, 5 mM MgCl_2_, 1 mg/mL lysozyme, and 5 units/mL DNase I). After sonication, inclusion bodies were collected by centrifugation. Then, the pellets were solubilized in buffer containing 6 M guanidine-HCl and 100 mM Tris-HCl pH 8.0. After centrifugation, the cleared lysate was obtained by filtration through a 0.45-µm filter, and the target protein was purified using a cOmplete™ His-tag purification column (Merck, Darmstadt, Germany) according to the manufacturer’s instructions. Denatured protein was mixed with 3 mg/mL NV10 (Novexin Ltd., Cambridge, UK) and refolded by sequential dialysis into the storage buffer supplemented with 50 mM Tris-HCl (pH 8.0) and 300 mM NaCl. Purification of the control His-SUMO protein was performed similarly as that of the HBV RT protein, except that the inclusion body-washing step was skipped, because the His-SUMO protein is a highly soluble protein.

### 2.3. Preparation of the Template/Primer-Bound Plates

Biotinylated poly(dA)_50_ or (rA)_50_ (5′-aaaa----aa-3′-Biotin) and oligo(dT)_8_ were synthesized by a manufacturer (Fasmac™, Atsugi, Kanagawa, Japan) and annealed at an equal mole ratio by heating at 65 °C for 5 min and immediate cooling down to 4 °C in a tris-buffered saline (TBS; 20 mM Tris-HCl pH 7.6, 150 mM NaCl). The annealed poly(dA)_50_/oligo(dT)_8_ (pdA/dT) or poly(rA)_50_/oligo(dT)_8_ (prA/dT) (100 pmol/well) was fixed on streptavidin-coated, 96-well plates (Thermo Fisher Scientific). One hundred µM desthiobiotin (Sigma), a stable analog of biotin, was added to mask unbound streptavidin after the binding of biotinylated poly(rA) or poly(dA)/oligo(dT) to the plates. After washing the plates twice with a washing buffer (25 mM Tris-HCl, pH 7.2, 150 mM NaCl, 0.1% BSA (bovine serum albumin), 0.05% Tween 20), they were dried and stored at 4 °C until use.

### 2.4. Template/Primer (T/P) Binding Assay

Purified HBV RT protein and a reaction buffer containing 50 mM Tris-HCl (pH 8.3), 75 mM KCl, 3 mM MgCl_2_, 10 mM DTT, and 40 units/mL RNase inhibitor (Merck) was added to the T/P-bound 96-well plates, as described above. After incubation of the plates at 37 °C for 3 h, each well was washed three times with 200 µL of a washing buffer (25 mM Tris-HCl (pH 7.2), 150 mM NaCl, 0.1% BSA, 0.05% Tween 20), and then 0.1 µg/mL of a mouse anti-His antibody (Nakalai Tesque, Kyoto, Japan) suspended in the washing buffer was added to the plates, which were then incubated for 1 h at room temperature. After the plates were washed three times with the washing buffer, 1:10,000 Horseradish Peroxidase (HRP)-conjugated polyclonal goat anti-mouse immunoglobulins (Dako, Santa Clara, CA, USA) was added, and the plates were again incubated for 1 h at room temperature. After the plates were washed, the binding activity of HBV RT with poly(rA)/oligo(dT) or poly(dA)/oligo(dT) was measured colorimetrically with an HRP substrate (sera care, Milford, MA, USA) at a wavelength of 405 nm using a microplate reader (GloMax^®^ Discover Multimode Microplate Reader, Promega, WI, USA). For the competition experiment of the T/P binding assay, either 40-fold or 100-fold of the non-biotinylated T/P was added to the reaction mixture. After incubation and washing the plates three times, an anti His-tag Ab was added. Activity was measured in a manner similar to that used for the T/P binding assay. At least three independent assays were performed for each experiment.

### 2.5. dUTP Substrate Binding Assay

The purified HBV RT (strep-RT-his8) protein was fixed on HisGrab™ copper-coated plates (PIERCE, California, USA) and the reaction buffer, which was the same as that for the T/P binding assay, and 10 µM fluorescein-12-dUTP (F-dUTP) (Merck) or 10 µM digoxigenin-11-2′-deoxy-uridine-5′-triphosphate (DIG-dUTP) (Merck) were added to the HBV RT-bound plates. After incubation at 37 °C for 3 h, each well was washed three times with the washing buffer. The fluorescent signal of dUTP bound with RT was measured by a microplate reader. For the detection of DIG-dUTP, 150 mU/mL of an anti-DIG-POD antibody (Merck) was added and the plates were incubated for an additional 90 min at room temperature. After the plates were washed with the washing buffer, the binding activity of RT with DIG-dUTP was detected by an ABTS colorimetric substrate. For the competition experiment, 10-, 50-, and 100-fold dTTP was added to the reaction mixture. For the inhibition assay, 75 pmol of HBV RT protein were fixed to copper-coated, 96-well plates. A total of 10 µM of compound suspended in DMSO and 10 µM of DIG-dUTP were mixed in the reaction mixture and added to the plates. Three independent assays were performed for each experiment.

### 2.6. Polymerase Binding Asay with Substrate and Template/Primer

Purified HBV RT and DIG-labeled dUTP were mixed in a reaction buffer containing 50 mM Tris-HCl pH 8.3, 75 mM KCl, 3 mM MgCl_2_, 10 mM DTT, and 40 units/mL RNase inhibitor, and the mixture was incubated on biotinylated T/P-bound, 96-well plates for 2 h at 37 °C. For the other polymerases, 40 units of superscript II (SS II), 2 units of T4 DNA polymerase, 0.1 units of Klenow fragment, and 0.1 units of Taq polymerase were used. EDTA was added at 50 mM to stop the reaction, and the plates were washed with the washing buffer three times. Then, an anti-DIG-POD antibody was added to the plates, which were incubated for 1 h at 37 °C. After washing the plates three times with the washing buffer, an ABTS colorimetric substrate was added to the plates, which were incubated for 30 min at 37 °C. The resultant activity was colorimetrically measured by wavelength absorbance at 405-nm. In this report, we referred to this assay as the “T/P and substrate binding assay”.

For the inhibition assay using the so-called “hit” compounds, 10 µM of compound were added to the T/P-bound plate before the addition of reaction mixture containing purified RT protein or another viral polymerase such as MMLV RT (Superscript II), T4 DNA polymerase, Klenow fragment, or Taq polymerase. The percentage of activity was calculated as follows: % Act = (X − NC)/(PC − NC) × 100; X, absorbance value of each sample; NC, absorbance value at 405 nm of a negative control (No RT or No Pol); PC, a positive control (DMSO; without compound).

### 2.7. Screening for Inhibitors of RT Protein-Specific Activity

Streptavidin-coated 384-well plates (Thermo Fisher Scientific) were used for HTS to find compounds with anti-RT activity. Biotinylated poly (rA)_50_/oligo (dT)_8_ templates (25 pmol/well) were bound to streptavidin-coated plates as described above. A total of 10 µM of compound and purified RT were incubated for 2 h at 37 °C in the reaction buffer containing DIG-dUTP. The reaction was stopped by addition of 50 mM EDTA. The following steps were performed in a manner similar to the protocol used for the detection of HBV RT polymerase, with the exception of the different buffer volume. For primary mass screening of compounds, all compounds that inhibited over 70% were considered first round hits (26/20,000 = 0.13%). For secondary screening, compounds that were filled as the selection standard to be active in the primary screening were further analyzed in a dose-dependent manner to validate hits. The compounds were used at 10 different concentrations (130, 43, 14, 4.8, 1.6, 0.5, 0.18, 0.06, 0.02, 0.007 µM). Three compounds that inhibited in a dose-dependent manner and showed under 10 µM of IC_50_ values were selected for the cell-based analysis.

### 2.8. Chemical Compounds

A library of pharmacologically active compounds (LOPAC^®^) containing 1280 compounds was purchased from Sigma. For the mass screening, small-molecule compound libraries (20,000 compounds) were obtained from the Center for Drug Discovery, Design, and Development at Osaka University. All chemical compounds were dissolved in dimethyl sulfoxide (DMSO) at 10 mM as a stock solution.

### 2.9. Cell-Based Analysis of Anti-HBV Agents

Candidate anti-HBV agents such as suramin, KB-R7943, and compound 3 were tested in cell-based assay systems, i.e., an HBV infection system using NTCP-expressing HepG2 (NTCP/G2) cells [16] and a stable HBV production system using HB611 cells, which was established by transfecting 3 tandemly arranged complete HBV DNA into a human hepatoblastoma cell line, Huh6 [17].

Drug cytotoxicity was tested for both cell systems. In the case of NTCP/G2 cells, cells were maintained in 100 µL PMM (primary hepatocyte maintenance media) containing 0.5 mg/mL G418 (PMM; William’s E Medium (Gibco, Grand Island, NY, USA)-10% FBS, 100 U/mL penicillin G, 100 µg/mL streptomycin, 0.25 µg/mL amphotericin B (Nakalai Tesque), 2 mM L-Glutamine (Nakalai Tesque), 5 µg/mL transferrin, 10 ng/mL EGF, 5 µg/mL insulin, 50 µM hydrocortisone, 5 µM dexamethasone, 5 ng/mL sodium selenite) (PMM-G418). The NTCP/G2 cells were seeded at 5 × 10^4^ cells/well on collagen-coated 96-well plates (IWAKI, Shizuoka, Japan) one day before starting the assay. The next day, the compounds were added to the medium with an additional 2% DMSO at the concentration shown in Figures 7 and 8. As regards the HB611 cells assay, the cells were maintained in the same PMM-G418 medium as used in the NTCP/G2 cells assay, and they were seeded at 500 cells/well on collagen-coated, 96-well plates one day before starting the assay. The drug-containing medium was exchanged every 3 days. Finally, viability was checked on the ninth day of the assay with CellTiter-Glo^®^ (Promega, WI, USA) according to the manufacturer’s instructions.

The effects of each compound on HBV production was basically assayed in the same manner with the exception of scale, i.e., in the case of the NTCP/G2 cells, 5 × 10^5^ cells/well were seeded on collagen-coated, 24-well plates (IWAKI), whereas 2 × 10^3^ cells/well HB611 cells were seeded on collagen-coated, 24-well plates upon initiation in 1 mL of PMM. Infection experiment in NTCP/G2 cells, 1000 GEI (genome equivalent of infection) of HBV obtained from HepAD 38.7 with PEG precipitation [16] was inoculated overnight with compounds. The compound-containing medium was exchanged every 3 days. On the final day (Day 9), the supernatant was collected after the cell debris had been excluded by spinning down the samples. The expression of HBsAg (hepatitis B surface antigen) and HBeAg (hepatitis B e-antigen) in the supernatants were measured by ELISA kits (HB S Antigen Quantitative ELISA Kit, Rapid-II (Beacle, Kyoto, Japan) for HBsAg; BJ Bioneovan HBeAg ELISA Kit (BJ Bioneovan, Beijing, China) for HBeAg). For the NTCP/G2 infection system, only HBeAg was measured, since a very high background was usually observed in the case of HBsAg. The collected supernatant was diluted by a factor of five.

Extracellular HBV DNA from the HB611 culture was prepared from the supernatant after assaying HBsAg and HBeAg. Briefly, to the harvested medium, PEG8000 was added at 10% as the final concentration, and the samples were left to stand still at 4 °C overnight. After centrifuge, pellets were suspended in Tris-buffered saline (20 mM Tris-HCl (pH 7.8), 150 mM NaCl) containing 5 mM MgCl_2_ and 10 units of DNase I (TAKARA BIO, Shiga, Japan) was added. The suspension was then incubated at 37 °C for 30 min to degrade contaminating cellular DNA. Then, EDTA (pH 8.0) was added at 10 mM to stop the reaction, with further inactivation of DNase I at 70 °C for 30 min. Both SDS (1.0%) and proteinase K (Merck) (0.2 mg/mL) were added to extract particle-associated HBV DNA by incubation at 56 °C overnight. The HBV DNA was finally prepared by phenol-chloroform-isoamyl alcohol (24:24:1) extraction followed by ethanol precipitation in the presence of 10 µg yeast tRNA and 25 µg glycogen (Nakalai Tesque).

Intracellular core-associated HBV DNA was basically prepared using the same methods as used in the extracellular HBV DNA preparation, except that the particles were extracted in a hypotonic buffer (20 mM Tris-HCl (pH 7.6), 50 mM NaCl, 5 mM MgCl_2_, 0.1% 2-mercaptethanol). After excluding the cell debris by centrifugation, non-particle-associated DNA was degraded with DNase I, and then the intracellular core-associated HBV DNA was extracted in the same manner as used for the extracellular HBV DNA preparation above.

Intracellular core-associated or extracellular particle-associated HBV DNA was extracted and evaluated by qPCR, the primers of which were set on the HBV S gene region (5′-CTTCATCCTGCTGCTATGCCT-3′ and 5′-AAAGCCCAGGATGATGGGAT-3′). The data were shown as HBV DNA copies/µL with a total volume of 20 µL.

To evaluate cccDNA formation, Hirt DNA was extracted from the compound-treated cells [18]. Briefly, the cells were lysed with TE (10 mM Tris-HCl pH 7.8, 1 mM EDTA pH 8.0)-1% SDS. RNA was degraded with 0.5 µg/mL RNase (Merck) at 65 °C for one hour and stood still at 4 °C overnight after adding NaCl at 0.5 M to precipitate high molecular weight DNA with SDS. Then, protease K (Merck) was added at 0.2 mg/mL and incubated at 56 °C overnight. After phenol-chloroform-isoamylalcohol extraction, DNA was precipitated with ethanol followed by drying up and was suspended in 100 µL of double distilled water (DDW) and 40 µL of the solution was treated with Plasmid-Safe™ ATP-Dependent-DNase (Epicentre, Madison, WI, USA) according to the manufacturer’s direction. The DNase was inactivated at 70 °C for 30 min. About 1 µL of the finally treated DNA was quantified for cccDNA by qPCR with cccDNA specific primers, forward; 5′-GTCTGTGCCTTCTCATCTGC-3′ and reverse; 5′-GCACAGCTTGGAGGCTTGAA-3′ as reported [19].

These experiments were performed at least three times, and the data are shown as the mean value with the standard deviation.

### 2.10. Statistical Analysis

All experiments were repeated at least three times with similar results. The results were recorded as the mean ± standard deviation (SD). Significance was defined as *p* < 0.05 using Welch’s *t*-test.

## 3. Results

### 3.1. Purification of Recombinant RT Proteins

**Figure 1:**
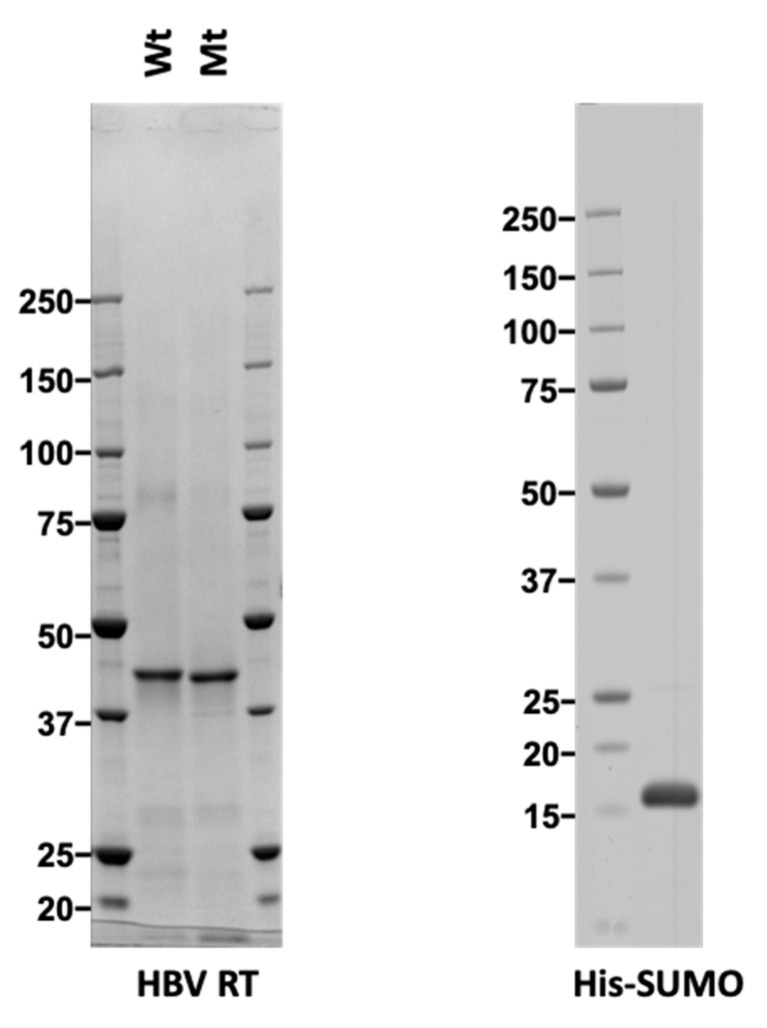
CBB staining of purified reverse transcriptase (RT) proteins. Wild type (Wt), and mutant (Mt) Hepatitis B virus (HBV) RT (**left** panel) proteins expressed in *E. coli* were purified under denaturing conditions and refolded. Control His-SUMO (Small Ubiquitin-like Modifier) (**right** panel) was also expressed in *E. coli* and purified under denaturing conditions. One microgram of each protein was separated by SDS-PAGE and stained with Coomassie Brilliant Blue (CBB).

We designed a recombinant RT protein with a strep-tag and an 8× His-tag at the N- and the C-terminus of the RT domain, respectively. Then we used a Ni-column purification system to purify the RT proteins expressed in *E. coli*. Because solubility, protein yield, and purity of the RT protein under non-denaturing conditions were very poor, the RT protein was purified under denaturing conditions. As a control, a mutant RT (in which the highly conserved YMDD motif at the active site had been exchanged to AAAA) was expressed and purified in the same manner as used for the wild-type RT. Subsequent Coomassie Brilliant Blue (CBB) staining of the purified wild-type and the mutant RT proteins showed that both were obtained with high purity (approximately >90%; Figure 1) at a similar purity level. Finally, mass spectrometry analysis was used to confirm that the purified protein was indeed HBV RT. A control protein, His-SUMO, was also expressed in *E. coli* and purified under denaturing conditions.

### 3.2. Purified RT Shows Specific T/P Binding Activity

**Figure 2:**
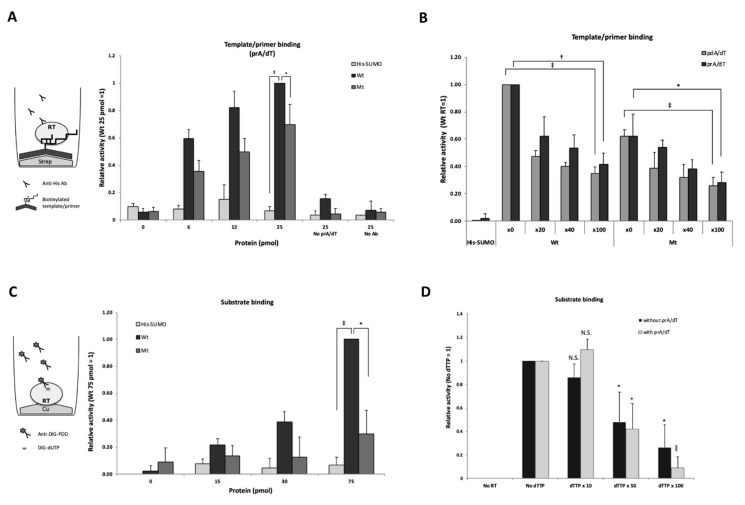
Purified RT showing specific poly(rA)/oligo(dT) and substrate binding activity. (**A**) poly(rA)/oligo(dT) (prA/dT) binding assay. Biotinylated prA/dT was fixed on a streptavidin-coated plate and 0, 6, 12, and 25 pmol of RT protein, respectively, were used for the reactions. Bound HBV RT to the template/primer was detected with an anti-His tag antibody. The value of 25 pmol of wild-type RT was arbitrarily set at 1, and relative values to this value were calculated. No prA/dT: assay without template/primer; No Ab: assay without the anti-His-tag antibody. (**B**) Competition assay of prA/dT binding. 20-fold, 40-fold, and 100-fold excess of either non-labeled prA/dT or pdA/dT was added to the reaction mixture containing HBV RT. Relative values compared to those lacking non-labeled prA/dT (x0) are shown. His-SUMO protein was used as a negative control. (**C**) Substrate binding assay. 0, 15, 30, and 75 pmol wild-type HBV RT and the mutant RT protein were fixed on a copper-coated plate. The value of 75 pmol wild-type HBV RT was arbitrarily set at 1, and the relative values are shown with the standard deviation. (**D**) Substrate binding competition analysis. Fifty pmol of wild-type HBV RT protein fixed on a copper-coated plate. For the substrate competition assay, respective non-labeled substrate (dTTP) (0, 10-, 50-, and 100-fold excess) was added to the reaction mixture containing 50 pmol of DIG-dUTP with or without 100 pmol of poly(rA)/oligo(dT). No RT; without RT protein. The value without competitor was set at 1, and the relative values are shown with the standard deviation. Statistical significance: *, *p* < 0.05; †, *p* < 0.01; ‡, *p* < 0.005; ǁ, *p* < 0.0005.

To determine whether or not the purified RT exhibited T/P binding activity, we performed a T/P binding assay using synthetic primer-templates, i.e., poly(dA)/oligo(dT) or poly(rA)/oligo(dT), respectively. Figure 2A shows the results of the poly(rA)/oligo(dT) binding assay in which the wild-type RT showed dose-dependent binding activity, while the mutant RT showed only modest activity.

To further confirm that the T/P binding activity was specific, a competition experiment was performed by the addition of non-biotinylated poly(rA)/oligo(dT) or poly(dA)/oligo(dT). As shown in Figure 2B, the poly(rA)/oligo(dT)- and the poly(dA)/oligo(dT)-binding activity of HBV RT competed with non-labeled poly(rA)/oligo(dT) and poly(dA)/oligo(dT), in a dose-dependent manner. Thus, the purified HBV RT exhibited specific binding activities with both poly(rA)/oligo(dT) and poly(dA)/oligo(dT).

### 3.3. Purified RT Shows Specific Substrate Binding Activity

To examine whether the purified RT had substrate binding activity, we performed a DIG (digoxigenin)-dUTP binding assay. Bound DIG-dUTP to the RT was detected by anti-DIG-POD antibodies with a luminescence substrate. As shown in Figure 2C, the activity of the wild-type RT had increased in a dose-dependent manner and this activity was significantly higher than that of the mutant RT and the control protein, His-SUMO. Thus, substrate binding activity appeared to be more specific for the wild-type HBV RT.

To further confirm that the substrate binding activity of RT was specific, a competition experiment was performed by adding non-labeled dTTP. After the addition of excess dTTP (10-, 50-, and 100-fold), the DIG-dUTP binding activity of RT decreased in a dose-dependent manner, both with or without poly(rA)/oligo(dT) (Figure 2D), which indicated that the purified RT had specific substrate binding activity. These results were not in conflict with previous reports showing that YMDD motif was more important for the substrate binding than template/primer binding as described in the discussion section [20,21].

### 3.4. Specific Binding Activity of Purified HBV RT Demonstrated by Cell-Free Assay

**Figure 3:**
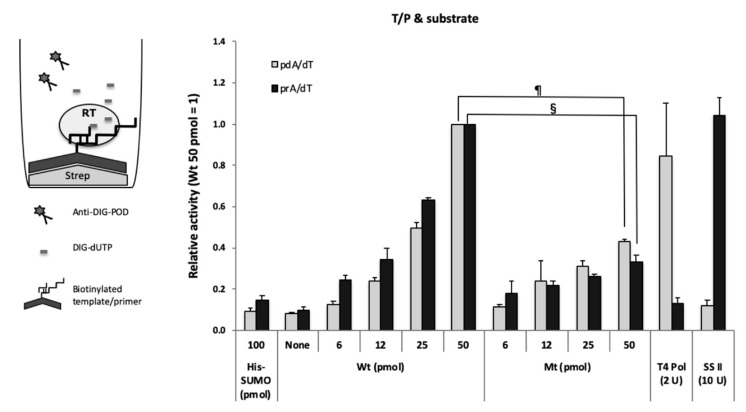
Template/primer and substrate binding assay. Biotinylated poly(dA)/oligo(dT) (dA/dT, gray bars) or poly(rA)/oligo(dT) (rA/dT, black bars) was fixed to streptavidin-coated plates. The value of 50 pmol wild-type HBV RT (Wt) was arbitrarily set at 1, and relative values are shown with the standard deviation. One hundred pmol of His-tagged SUMO (His-SUMO) were used as a negative control. Two units of T4 DNA polymerase and ten units of SS II were used as the positive controls for DNA template-dependent and RNA template-dependent activity, respectively. None; no HBV RT protein. §, *p* < 0.001; ¶, *p* < 0.0001.

To examine whether the purified HBV RT exhibited polymerase activity, we constructed an in vitro polymerase assay using a substrate, DIG-dUTP, and a synthetic primer-bound template, i.e., poly(rA)/oligo(dT) or poly(dA)/oligo(dT) (Figure 3). The purified RT appeared to exhibit the activity in a dose-dependent manner (Figure 3, wild-type RT (Wt)). To examine the specificity of the RT activity, we tested the activity of the mutant RT. The activity of the mutant RT was lower than that of the wild-type RT (Figure 3, mutant RT (Mt)) in both cases, i.e., poly(dA)/oligo(dT) and poly(rA)/oligo(dT). However, no replication products of HBV RT were detected using acrylamide gel electrophoresis after the reaction, although replication products of MMLV RT were detected (data not shown). In fact, both wild-type and mutant HBV RT showed template/primer binding activity, with slightly less activity in the case of the mutant RT (Figure 2A,B). On the other hand, greater substrate binding activity was observed in the case of the wild-type HBV RT (Figure 2C). Therefore, we concluded that purified HBV RT proteins exhibited both of T/P and substrate binding activities but not elongation activity in this assay (i.e., T/P & substrate binding assay) (Figure 3).

In this study, T4 DNA polymerase showed high polymerase activity only in the case of poly(dA)/oligo(dT) with substrates, but not in the case of poly(rA)/oligo(dT) (Figure 3), and the reverse was observed with MMLV RT (SS II in Figure 3).

### 3.5. Pilot Screening for HBV RT Inhibitors with Purified HBV RT

Although our purified HBV RT did not show elongation activity, it was possible to establish an HBV RT activity detection system with a T/P and substrates to find new anti-HBV agents, since the HBV RT showed T/P-binding and substrate-binding activity, as shown in Figure 2. To examine whether this assay system was available for the screening, we firstly tested 100 µM compound concentration for the pilot screening. We used a library of pharmacologically active compounds (LOPAC^®^) for searching candidate inhibitors. LOPAC^®^ is a collection of chemical compounds in which the structure and function have been known (please see the manufacturer’s web site <https://www.sigmaaldrich.com/life-science/cell-biology/bioactive-small-molecules/lopac1280-navigator.html> for the details).

**Figure 4:**
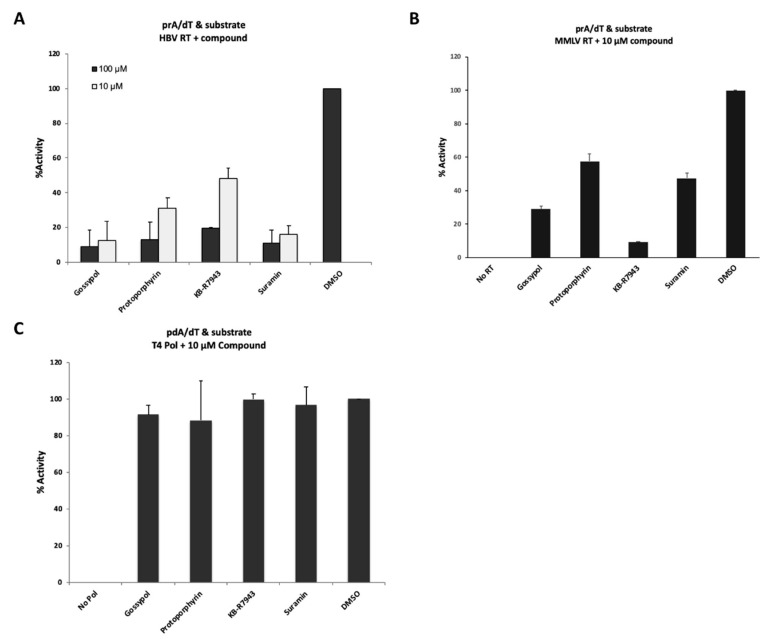
The evaluation of inhibitory effects by “hits” from the LOPAC^®^ library. (**A**) Inhibitory effect of “hit” compounds on a template/primer and substrate binding activity of HBV RT. 10 µM (light gray bar) or 100 µM (black bar) compound were added to the reaction mixture. (**B**) Inhibitory effect of “hit” compounds on MMLV RT activity. Forty units of SS II were added to the reaction mixture containing each 10 µM compound. No RT, no SS II with 6% DMSO; DMSO, 6% DMSO with SS II. (**C**) Evaluation of inhibitory effects of “hit” compounds on T4 DNA polymerase. Two units of T4 DNA polymerase were added to the reaction buffer containing each 10 µM compound. No Pol, no T4 DNA polymerase with 6% DMSO; DMSO, 6% DMSO with T4 DNA polymerase (control).

Among 1280 compounds, we found 26 compounds showing a relatively high inhibitory effect (% inhibition, >90%) at 100 µM (data not shown). Some of these compounds have already been reported as viral replication inhibitors: suramin [22,23,24,25,26,27,28,29,30], NF023 [27], gossypol [31,32,33], rottlerin [34,35], and reactive blue 2 [36], and they inhibited our purified HBV RT as well, in a dose-dependent manner (Table 1 and Figure 4A). Interestingly, suramin and NF023 were identified as inhibitors of human Norovirus RNA-dependent RNA polymerase (RdRp) by an in silico docking search, and its binding site was located between the RdRp fingers and thumb domains, based on the three-dimensional structure of several viral polymerases with compounds reported recently [27]. Suramin and its derivatives were reported to inhibit the polymerase activity of duck hepatitis B virus (DHBV), a hepadnavirus family [28,37], and that of other retroviruses, including human T-cell lymphotropic virus (HTLV-1) [26] and human immunodeficiency virus-1 (HIV-1) [23].

To identify the compounds that had the higher inhibitory effect, the compounds were retested at 10 µM. Four compounds (gossypol, protoporphyrin IX disodium, KB-R7943, and suramin sodium salt) showed the higher inhibitory effects, even at 10 µM in the “T/P and substrate” binding assay (Figure 4A). To examine whether or not these four compounds could inhibit other types of viral RT activity, we used MMLV RT (SuperScript II, SS II) for the inhibition assay (Figure 4B). Four compounds showed varying levels of inhibition of SS II activity, although their inhibition efficiency could not be directly compared with that of HBV RT, since SS II also possesses elongation activity. Interestingly, the compounds selected in this manner did not inhibit the activity of T4 DNA polymerase, which requires poly(dA)/oligo(dT) as a template/primer (Figure 4C).

Taken together, these results suggest that the present assay system could be useful for screening for candidate inhibitors, which prevented a template/primer and/or substrate binding activity of HBV RT.

### 3.6. Mass Screening for Novel Hit Compound

Furthermore, we tried to screen an additional small molecule compound library obtained from the Center for Drug Discovery, Design, and Development at Osaka University. Based on the hit rate (~0.5%), we used 10 µM of compounds for the screening. After testing repeatedly, we obtained four hits (compound 2–5) as putative inhibitors of the HBV RT from among the 20,000 small molecule compounds of the library.

**Figure 5:**
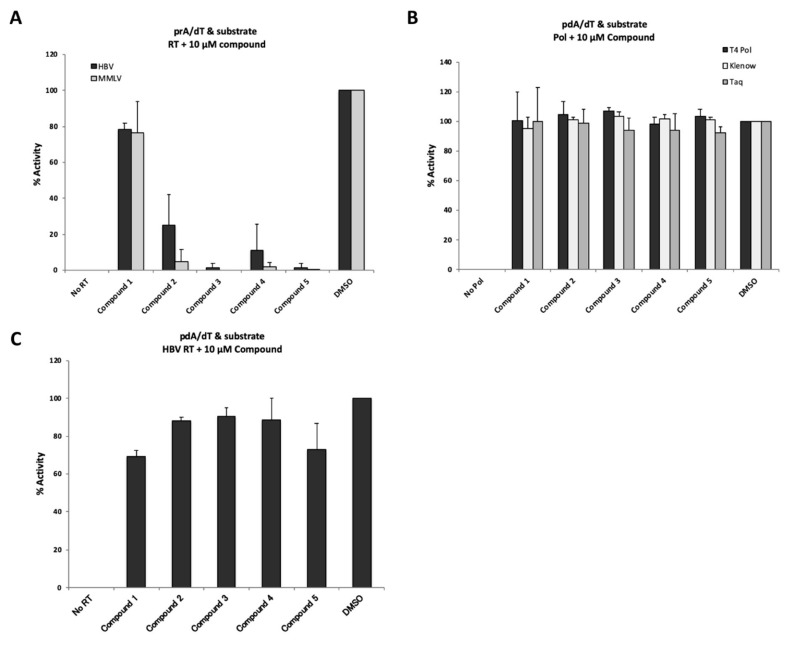
Evaluation of inhibition by “hit” compounds identified via mass screening. (**A**) Inhibitory effects of “hit” compounds on HBV RT and MMLV RT activity. Poly(rA)/oligo(dT) was used as a template/primer. Ten µM of each compound was added to the reaction mixture. (**B**) Evaluation of inhibitory effects of “hit” compounds on DNA polymerases. Poly(dA)/oligo(dT) was used as a template/primer. No Pol; no DNA polymerase with 6% DMSO, DMSO; 6% DMSO with DNA polymerase. (**C**) Modest inhibitory effect of “hit” compounds on DNA template-dependent activity of HBV RT. The detection assay was performed according to a similar protocol as that employed for the experiment shown in Figure 5A, except for the use of poly(dA)/oligo(dT)-coated plates. No RT, no HBV RT protein with 6% DMSO; DMSO, 6% DMSO with HBV RT protein. The data are shown as mean values with the standard deviation.

To investigate whether the four putative inhibitory compounds indeed exerted inhibitory effects on the polymerase activity of other species such as MMLV RT, T4 DNA polymerase, Klenow fragment, and Taq polymerase, we examined the effects of each compound at 10 µM on the activity of these polymerases (Figure 5). As shown in Figure 5A, the four putative inhibitory compounds numbered 2, 3, 4, and 5 identified from the small molecule library did significantly inhibit the RT activity of MMLV RT, as well as that of HBV RT. These results suggested that the present system should be useful for discovering MMLV RT inhibitors, although our screening system was intended to identify only T/P and substrate binding inhibitors, but not elongation inhibitors. On the other hand, none of the four hit compounds inhibited the activity of DNA-dependent DNA polymerases such as T4 DNA polymerase, Klenow fragment and Taq polymerase (Figure 5B). Since HBV Pol has DNA-dependent as well as RNA-dependent DNA synthesis activity, we examined its inhibitory effects on DNA-dependent DNA synthesis activity using the poly(dA)/oligo(dT) as the template/primer. Interestingly, these four putative inhibitory compounds at 10 µM did not show any inhibitory effects on the activity (i.e., poly(dA)/oligo(dT) with substrates) of the HBV RT, although compound 5 inhibited 20 to 30% of the control (DMSO) (Figure 5C).

### 3.7. Four New Putative Inhibitory Compounds Identified Exhibited RNA Template-Specific Inhibition

**Figure 6:**
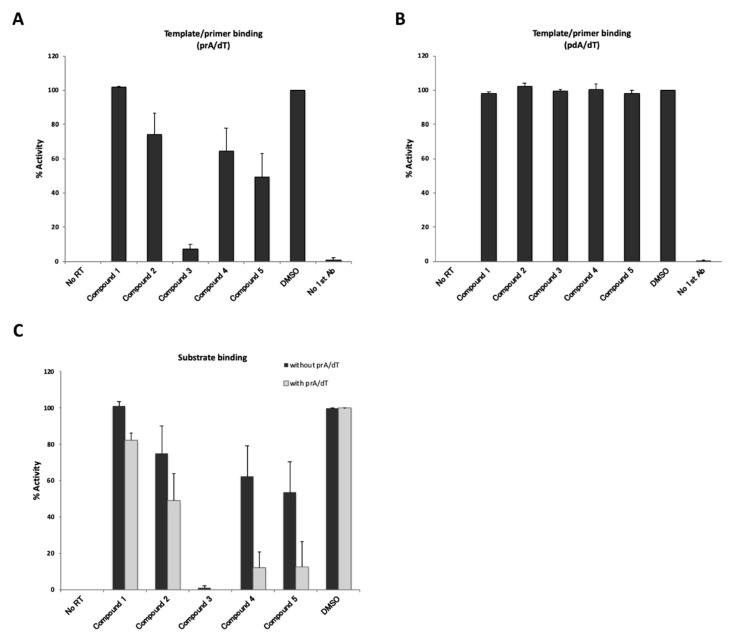
Evaluation of inhibitory effects of “hit” compounds on template/primer or substrate binding activity. No RT; no HBV RT protein with 6% DMSO, DMSO; 6% DMSO with HBV RT protein. (**A**) Inhibition of poly(rA)/oligo(dT) binding activity of HBV RT. Ten µM of compound were mixed with the reaction buffer and added to poly(rA)/oligo(dT)-coating plates. (**B**) Inhibition of poly(dA)/oligo(dT) binding activity of HBV RT. Ten µM of compound were mixed with the reaction buffer and added to poly(dA)/oligo(dT)-coating plates. (**C**) Inhibition of “hit” compounds on the substrate binding activity of HBV RT. 75 pmol of HBV RT were fixed to copper-coated plates, and 10 µM of each compound and 10 µM DIG-dUTP were added to the mixtures, which were then incubated for 3 h in the presence (with prA/dT, gray bars) or absence (without prA/dT, black bars) of the template/primer. The data are shown as mean values with the standard deviation.

To determine whether these newly identified putative inhibitory compounds were indeed able to inhibit the T/P and/or substrate binding activity of HBV RT, we performed T/P binding assays with either poly(rA)/oligo(dT) or poly(dA)/oligo(dT) (Figure 6A,B). As shown in Figure 6A, compounds 2, 3, 4, and 5 inhibited poly(rA)/oligo(dT) binding activity of HBV RT at the following respective percentages: 74%, 7%, 64% and 49%. On the other hand, these compounds did not have any effect on poly(dA)/oligo(dT) binding activity (Figure 6B). These results suggest that the inhibitory effects of these compounds on HBV RT activity should be dependent on the RNA template.

We then examined the inhibitory effects of the hit compounds on the substrate binding activity of HBV RT (Figure 6C). Although the inhibitory effects were found to be weaker without a template, except for in the case of compound 3 (Figure 6C), the inhibition tendencies of the substrate binding activity were similar to the results of the poly(rA)/oligo(dT) binding (Figure 6A). Either with or without the poly(rA)/oligo(dT), compound 3 significantly inhibited RT-dUTP substrate binding activity. In contrast, compounds 4 and 5 inhibited substrate binding activity more effectively in the presence of poly(rA)/oligo(dT) (Figure 6C, with prA/dT). This result seemed to be reasonable, because both assays shown in (Figure 5A and (Figure 6C “with poly(rA)/oligo(dT)”, reflected primarily substrate binding activity in the co-presence of poly(rA)/oligo(dT). From these results, our screening system should be expected to be useful for identifying inhibitors of template/primer and substrate binding activity of HBV RT.

### 3.8. Cell-Based Assay for Hit Compounds

Then, the newly identified hit compounds were subjected to cell-based assay using an NTCP-expressing HepG2 cell line (NTCP/G2) as an HBV infection system and HB611 cells as an artificial HBV production system [17,38,39,40,41,42,43]. Not all of the putative inhibitory hit compounds showed inhibition of HBV DNA levels (data not shown). However, some of these compounds did appear to be effective in reducing HBV DNA levels. We tested these potential compound efficacies and cytotoxicity in NTCP/G2 and HB611 cell systems and calculated a 50% cytotoxic concentration (CC_50_) and putative 50% inhibition concentration (IC_50_) and then selectivity index (SI = CC_50_/IC_50_), though a wider range of drug concentration for assay should be required to determine accurate IC_50_.

**Figure 7:**
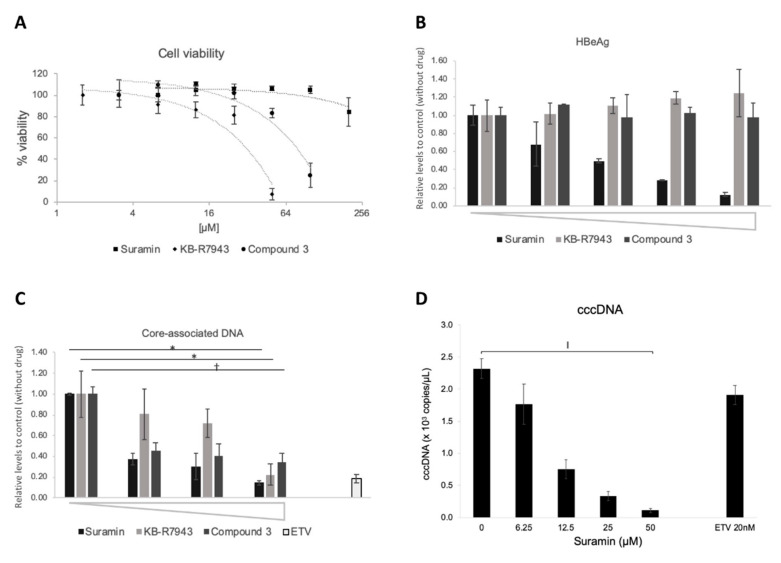
Inhibitory effects of HBV DNA levels by compounds in NTCP/G2 cells. Effect of compounds on HBV DNA levels was evaluated in the NTCP/G2 infection system. (**A**) Cytotoxic analysis of suramin (■), KB-R7943 (◆), and compound 3 (●) on NTCP/G2 cells. % of viability is shown as ratio to the control (no compound). The horizontal axis is shown as log_2_ scale. Compound concentration of suramin: 6.3, 12.5, 25, 50, 100, 200 µM; KB-R7943: 1.6, 3.1, 6.3, 12.5, 25, 50 µM; compound 3: 3.1, 6.3. 12.5, 25, 50, 100 µM. (**B**) The effect on HBeAg (hepatitis B e-antigen) production by compounds. The culture supernatant was collected on the last day of the assay and was subjected to HBeAg ELISA, as described in Materials and Methods. Compound concentration of suramin: 0, 6.3, 12.5, 25, 50 µM; KB-R7943: 0, 1.3, 2.5, 5, 10 µM; compound 3: 0, 3.1, 6.3, 12.5, 25 µM. (**C**) Core-associated HBV DNA production. Intracellular HBV core-associated DNA was extracted and evaluated by qPCR. Compound concentration of suramin: 0, 12.5, 25, 50 µM; KB-R7943: 0, 2.5, 5, 10 µM; compound 3: 0, 1.6, 3.1, 6.25 µM; Entecavir: 20 nM. *, *p* < 0.05; †, *p* < 0.01. (**D**) The effect on cccDNA levels by suramin. Cells were treated with 0, 6.3, 12.5, 25, and 50 µM of suramin. ǁ, *p* < 0.0005.

**Figure 8:**
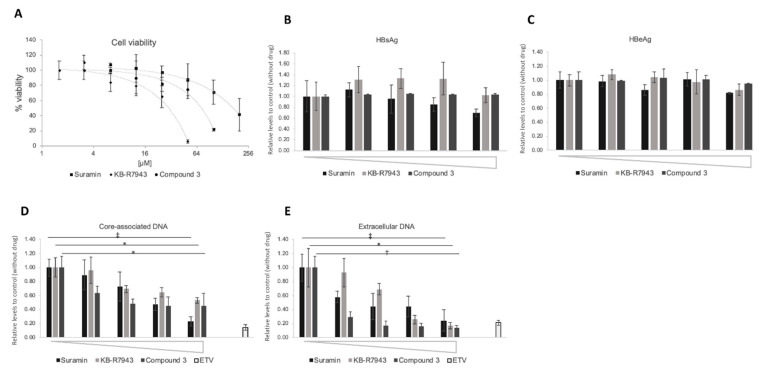
Inhibition of HBV DNA levels by compounds in HB611 cells. The effect of compounds on HBV DNA levels was evaluated in HB611 cells stably producing HBV. (**A**) Cytotoxic analysis of suramin (■), KB-R7943 (◆), and compound 3 (●) on HB611 cells. % of viability is shown as ratio to the control (no compound). The horizontal axis is shown as log_2_ scale. Compound concentration of suramin: 6.3, 12.5, 25, 50, 100, 200 µM; KB-R7943: 1.6, 3.1, 6.3, 12.5, 25, 50 µM; compound 3: 3.1, 6.3. 12.5, 25, 50, 100 µM. (**B**) (**C**) The effect on HBsAg (hepatitis B surface antigen) and HBeAg production by compounds. The culture supernatant was collected on the last day of the assay and was subjected to HBsAg (**B**) and HBeAg (**C**) ELISA, as described in Materials and Methods. Compound concentration of suramin: 0, 12.5, 25, 50, 100 µM; KB-R7943: 0, 2.5, 5, 10, 20 µM; compound 3: 0, 3.1, 6.3. 12.5, 25 µM. Core-associated (**D**) and extracellular particle-associated HBV DNA (**E**) production. Compound concentration of suramin: 0, 12.5, 25, 50, 100 µM; KB-R7943: 0, 1.3, 2.5, 5, 10 µM; compound 3: 0, 1.3, 2.5, 5, 10 µM; Entecavir: 20 nM. Intracellular core-associated (D) or extracellular particle-associated (**E**) HBV DNA were extracted and evaluated by qPCR. *, *p* < 0.05; †, *p* < 0.01; ‡, *p* < 0.005.

Suramin and KB-R7943 were identified from LOPAC^®^ chemical library as an HBV RT inhibitor in the “T/P and substrate” binding assay (Figure 4A,B), and compound 3 showed highest inhibition activity against poly(rA)/oligo(dT) templates as well as against substrate binding (Figure 5A and Figure 6A,C). As shown in (Figure 7A and Figure 8A, CC_50_ values of suramin, KB-R7943, and compound 3 were >200, 37.4, 66.9 µM (in NTCP/G2) and 168, 41.9, and 72.6 µM (in HB611), respectively. In the case of the KB-R7943 and compound 3, HBeAg expression in the culture supernatant did not change in NTCP/G2 (Figure 7B), which indicated that the drug blocked neither HBV attachment/entry, nor transcription. In contrast, suramin reduced HBeAg and cccDNA levels in NTCP/G2 cells (Figure 7B,D). All three compounds decreased the amount of core-associated HBV DNA in a dose-dependent manner (Figure 7C). The putative IC_50_ of suramin, KB-R7943, and compound 3 in NTCP/G2 were calculated to be 0.27, 7.37, and 0.12 µM, respectively.

In the HB611 system, no changes in HBsAg and HBeAg secretion were observed in amounts of suramin, KB-R7943, and compound 3 up to 50, 20, and 25 µM, respectively (Figure 8B,C). Core-associated and extracellular HBV-related DNA were reduced in a dose-dependent manner (Figure 8D,E). The IC_50_ of suramin, and compound 3 for core-associated DNA were 26.4 and 1.31 µM, respectively, although KB-R7943 showed a modest decrease (Figure 8D). The IC_50_ of suramin, KB-R7943, and compound 3 for extracellular HBV production were calculated to be 16.8, 3.7, and 0.21 µM, respectively (Figure 8E).

## 4. Discussion

Here, we developed an in vitro assay system to find potential RT inhibitors using purified HBV RT. We focused on expressing the RT domain of HBV Pol in *E. coli* and successfully obtained highly pure HBV RT. The purified RT protein exhibited T/P and substrate binding activity (Figure 2). With mutation in the YMDD, the substrate binding activity was significantly lost, but not that of T/P binding (Figure 2A,C). These results were not in conflict with those of previous studies in that the YMDD motif of HIV-1 RT was found to be more important for substrate binding than was T/P binding, and the WMGY motif proximal to YMDD played a more important role in primer grip [20,21]. Since these motifs are highly conserved in HBV and duck HBV (DHBV) [21], it is likely that the YMDD mutant possesses less substrate binding than T/P binding activity. However, it is probable that the T/P binding activity is somewhat affected by the YMDD mutation, because the YMDD domain is proximal to the primer grip domain, WMGY.

We used these findings to establish the present system for screening anti-HBV agents and were able to find several candidate inhibitors against the HBV RT, several of which had been suggested previously as anti-viral polymerase agents (Table 1). It is suggested that a part of RNase H domain should be required for the elongation activity of HBV RT (unpublished data from other researchers). However, “T/P and substrate” binding assay is thought to be available for finding candidate NNRTIs. Both T/P binding and substrate binding are common crucial steps required by viral polymerases, including HBV Pol [14,15]. Therefore, functional domains that interact with the T/P and incoming dNTPs are highly conserved among the RTs of other species [9,10,11,12,13]. Furthermore, several of the hit compounds in this study were found to inhibit the activity of MMLV RT (SS II), which has polymerizing/elongation activity (Figure 4B and Figure 5A). We found that these identified compounds showed similar inhibitory effects, both on HBV RT and MMLV RT activity (Figure 4A,B and Figure 5A).

Generally, polymerases contain highly conserved domains. Accordingly, they can be separated into seven families, A, B, C, D, X, Y, and RT, based on their sequence homology [44,45,46,47,48]. The T4 DNA polymerase derived from the T4 phase belongs to family B, a type similar to human DNA polymerase α, δ, and ε [44,45,46,47]. Klenow fragment derived from *E. coli* and Taq polymerase derived from Thermus aquaticus belong to family A, which is similar to mitochondrial DNA polymerase γ [48]. Interestingly, the inhibitory effects of these compounds on poly(dA)/oligo(dT) and poly(rA)/oligo(dT) clearly differed (Figure 4 and Figure 5). The hit compounds did not significantly inhibit the DNA template-specific binding activity of HBV RT (Figure 5C), nor did they inhibit other types of DNA polymerase such as T4 DNA polymerase, DNA polymerase I (Klenow), or Taq polymerase (Figure 4C and Figure 5B). These compounds exerted no effect on the poly(dA)/oligo(dT) binding activity of HBV RT (Figure 6B), and thus the hit compounds identified here specifically targeted only the viral RNA templates, but not the DNA templates, which are those for human DNA polymerase. These differences can be accounted for by the structural differences between RNA/DNA and DNA/DNA duplexes [20,49,50,51,52,53] and the divergent binding kinetics of RNA/DNA versus DNA/DNA template/primer to HIV-1 RT [20,53].

This study demonstrated that chemicals nominated via our “T/P and substrate” binding assay, namely, suramin, KB-R7943, and compound 3, showed inhibitory activity against HBV replication. Suramin had been known to be an inhibitor of various enzymes, including Trypanosoma glycolytic enzymes [54,55], and viral and cellular DNA and RNA polymerase [56]; it had been also known as an inhibitor of the RT of RNA tumor viruses [26,57]. As regards HBV, suramin was reported to exert inhibitory effects on duck HBV RT [28,37]. Indeed, this drug is of particular interest due to its potential for inhibiting the HBV life cycle at various critical stages. For example, suramin inhibits PKA-mediated HBV core phosphorylation [58,59], and it also interferes with HBV entry into the cell by inhibiting a purinergic receptor [60], in addition to its anti-HBV RT activity. In our assay, suramin was found to exert a direct inhibitory effect on HBV RT (Figure 4A). In an assay using an HBV infection system (NTCP/G2 cells), not only HBV DNA, but also HBeAg and cccDNA levels were reduced by suramin, suggesting that HBV entry was also remarkably inhibited (Figure 7). Furthermore, in a stable HBV production system (HB611 cells), levels of HBV DNA were clearly reduced without any observable effect on HBsAg or HBeAg production (Figure 8). Because the HBV production system does not have the entry step, suramin is likely to inhibit HBV DNA amplification. As reported, suramin is not likely a specific HBV inhibitor [26,28,37,57]. However, the inhibitory effects of suramin on HBV could be not only at the HBV DNA amplification steps, but also many steps including HBV entry [60].

Our screening system using the RT domain also identified the protoporphyrin IX disodium as a hit compound (Figure 4). A previous study reported that porphyrin compounds including protoporphyrin IX disodium suppressed the protein-priming reaction [61]. In that report, the authors demonstrated that these compounds could target the terminal protein (TP) domain of the polymerase. However, the RT domain alone could also partially rescue the protein priming activity of DHBV MiniRT2 protein, which contains TP and RT domains, in the presence of the iron protoporphyrin IX (Hemin), suggesting that not only TP domain, but also RT domain alone might have another hemin binding site [61]. Our study supports the possibility that protoporphyrin IX disodium could indeed interact with RT and inhibit its activity.

This is the first report to demonstrate the inhibitory effect of KB-R7943 on HBV DNA replication. KB-R7943 is an inhibitor of Na^+^/Ca2^+^ exchanger (NCX), a major regulator of the intracellular calcium concentration in various cell types [62]. The inhibitory effect on HBV replication by NCX suggests that this kind of signaling could be important for HBV replication in hepatocytes.

Compound 3 was found in the chemical library of the Center for Drug Discovery, Design, and Development at Osaka University. This drug specifically inhibited RNA-based T/P binding (prA/dT), as well as substrate binding, but not DNA-based T/P binding (Figure 6). This result was consistent with the observed lack of inhibition by compound 3 of the “poly(dA)/oligo(dT) and substrate” binding activity (Figure 5C). This result indicates that compound 3 participates in the reverse transcription (RNA template-dependent DNA replication) process. Compounds 2, 4, and 5 moderately inhibited the binding of poly(rA)/oligo(dT) alone with RT (Figure 6A) or substrate alone with RT (Figure 6C; without prA/dT), but they were more effective in inhibiting substrate binding when in the co-presence of poly(rA)/oligo(dT) (Figure 6C; with prA/dT). Considering that these compounds inhibited substrate binding in the co-presence of a poly(rA)/oligo(dT), it was assumed that they interfered with the uptake of dNTPs into the RT that was bound to the poly(rA)/oligo(dT). Compound 3 clearly inhibited HBV replication in the cell-based assays conducted here. Since the drug did not inhibit the expression of HBsAg or HBeAg, it is likely that compound 3 inhibits the reverse transcription of HBV replication, although it is not able to exclude the possibilities that hit compounds could have effects on other domains of polymerase such as TP and RNase H domain, and/or the capsids which are required for viral RNA packaging and DNA synthesis. Since hit compounds were obtained as inhibitors of RT protein-specific activity, it would be reasonable that such compounds exhibited the overall same inhibition activity in a cell-based assay, however, the details of the mechanism of action in cell-based assays must be clarified by using an endogenous polymerase assay. The putative selectivity index (SI = CC_50_/IC_50_) of compound 3 for the inhibition of intracellular core-associated HBV DNA formation was 558 and 55 in NTCP/G2 and HB611 cells, respectively, and the SI value for the extracellular particle-associated HBV DNA was 353 in HB611 cells (Figure 7 and Figure 8). Thus, further chemical optimization of this compound is expected to lead to the generation of a novel anti-HBV agent; needless to say, more precise analysis will still be required to elucidate relevant mechanisms of action. Further investigation of these drugs’ mechanism of action will facilitate the development of innovative anti-HBV drugs.

The non-nucleos(t)ide reverse transcriptase inhibitors (NNRTIs), which bind to the allosteric pocket of HIV RT, do not directly inhibit the binding of the dNTP of RT [63], although the molecular mechanisms of NNRTI inhibition have not yet been elucidated. However, NNRTIs generally show high potency, selectivity, and the lower cell toxicity [64,65,66,67]. It is expected that such NNRTIs could be identified by our screening system, because compounds found in the present system showed template/primer- and substrate-binding inhibitory effects as those of the NNRTIs. A recent study has demonstrated that drug combination treatment using NRTIs and NNRTIs showed the stronger synergistic inhibitory effect on infection by HIV-1 strains, including those resistant to NRTIs [7]. Note that, in the clinical setting, the hits from this study may not reduce the incidence of HBV-related diseases; further chemical modification of compounds and assessment of the effects and cytotoxicity of these compounds using animal model systems will be necessary, as investigations into their particular mechanisms of action.

To overcome HBV-related diseases, elimination of cccDNA is known to be critical. Many researchers have proposed new strategies to eliminate cccDNA; for example, genome editing [68,69,70,71,72,73]. Such methods, however, seem to take much time for clinical use and multi-drug combination therapy targeting various steps of the viral life cycle should be a more realistic strategy to treat the HBV-infected patients. Our developed screening system should be valuable for developing novel drugs targeting HBV polymerase.

## 5. Conclusions

In this study, we successfully obtained a highly pure HBV RT, which enabled us to develop a novel HTS assay for finding HBV polymerase inhibitors and also open the door to clarify the crystal structure of HBV RT protein. This is the first report to demonstrate a high-throughput screening using the purified RT protein. Our high-throughput screening system will facilitate the exploration for novel RT inhibitors against HBV and contribute to the treatment of HBV-infected patients.

## Figures and Tables

**Table 1:** A summary of already known anti-viral polymerase agents identified in the present screening assay.

Compound	% Inhibition ^1^		Target Viruses ^2^	Refs.
	100 µM	10 µM		
Suramin	97	69	HTLV, DHBV, HIV-1, DENV, RVFV, NV, EV71, encephalitis B virus, CHIKV	[20,21,22,23,24,25,26,27,28]
NF023	96	26	NV	[25]
Gossypol	97	92	HSV-2, HIV-1, Influenza virus	[29,30,31]
Rottlerin	98	35	HIV-1	[32,33]
Reactive blue 2	100	53	HCMV	[34]

^1^ % inhibition to HBV RT of cell-free assay. ^2^ Target viruses were abbreviated: HTLV, Human T-lymphotropic virus; DHBV, Duck hepatitis B virus; HIV-1, Human immunodeficiency virus-1; DENV, Dengue virus; RVFV, Rift Valley fever virus; NV, Norovirus; EV71, Enterovirus 71; CHIKV, Chikungunya virus; HSV-2, Herpes simplex virus 2; HCMV, Human cytomegalovirus.
